# Hemoglobin-Geriatric Nutritional Risk Index Predicts Major Adverse Cardiovascular Events After Transcatheter Aortic Valve Implantation

**DOI:** 10.3390/nu17213419

**Published:** 2025-10-30

**Authors:** Takeshi Sasaki, Takahiro Miura, Harutoshi Tamura, Yuya Takakubo, Michiaki Takagi, Satoru Ebihara

**Affiliations:** 1 Department of Rehabilitation Medicine, Tohoku University Graduate School of Medicine, Sendai 980-8574, Japan; takeshi.sasaki.t3@dc.tohoku.ac.jp (T.S.); takahiro.miura.d1@tohoku.ac.jp (T.M.); 2 Rehabilitation Unit, Yamagata University Hospital, Yamagata 990-9585, Japan; takakubo-y@med.id.yamagata-u.ac.jp (Y.T.); mtakagi@med.id.yamagata-u.ac.jp (M.T.); 3 Department of Cardiology, Pulmonology, and Nephrology, Yamagata University School of Medicine, Yamagata 990-9585, Japan; htamura@med.id.yamagata-u.ac.jp; 4 Department of Orthopaedic Surgery, Faculty of Medicine, Yamagata University, Yamagata 990-9585, Japan

**Keywords:** nutritional status, malnutrition, anemia, transcatheter aortic valve replacement

## Abstract

**Background/Objectives**: Numerous older patients undergo transcatheter valve implantation (TAVI) and frequently experience preoperative malnutrition and anemia, which markedly influence postoperative outcomes. This study investigated whether the Hemoglobin-Geriatric Nutritional Risk Index (H-GNRI) could predict major adverse cardiovascular events (MACEs) after TAVI. **Methods**: Patients who underwent TAVI at a single institution were classified into three groups according to their H-GNRI scores: low-risk (H-GNRI score = two), intermediate-risk (H-GNRI score = one), and high-risk (H-GNRI score = zero). The primary outcome was the occurrence of MACEs post-TAVI, and Kaplan–Meier survival and Cox proportional-hazard analyses were performed. **Results:** Of the 205 patients analyzed, 123, 67, and 15 were assigned H-GNRI scores of two, one, and zero. Kaplan–Meier survival analysis revealed that patients with H-GNRI scores of one and zero developed significantly more MACEs than those with a score of two (log-rank *p* = 0.0030; 1 vs. 2, *p* = 0.0032; 0 vs. 2, *p* = 0.0077). In the Cox proportional-hazard analysis, factors associated with MACEs included H-GNRI score (using score two as reference; score one: hazard ratio [HR] = 2.02, 95% confidence interval [CI] = 1.10–3.60, *p* = 0.021; score 0: HR = 2.67, 95% CI = 1.10–6.44, *p* = 0.028), procedure time (HR = 1.00; 95% CI = 1.00–1.01; *p* = 0.0093), and length of hospital stay after TAVI (HR = 1.02; 95% CI = 1.01–1.04, *p* = 0.0003). **Conclusions**: Preoperative H-GNRI scores were markedly associated with the incidence of postoperative MACEs in patients undergoing TAVI.

## 1. Introduction

Transcatheter aortic valve implantation (TAVI) is a minimally invasive procedure that imposes a low burden on patients with aortic stenosis (AS). It can be performed in older patients and those with high surgical risk, and its target patient population has expanded in recent years with further widespread adoption expected [1,2,3]. Although TAVI has been associated with favorable postoperative outcomes, postoperative rehospitalization, mortality risk, and major adverse cardiovascular events (MACEs) remain clinical challenges. Therefore, risk stratification and early intervention are crucial to improving outcomes [4]. With the current trend of expanding indications for lower-risk patients, identifying the risk of MACEs is necessary to improve prognosis [5]. Surgical risk is usually assessed by employing surgical risk scores prior to TAVI. However, for patients with multiple comorbidities undergoing TAVI, the inclusion of nutritional and frailty indicators in risk assessments has been shown to improve the prediction of long-term mortality, underscoring the importance of composite indicators [6].

Numerous patients who undergo TAVI are older and often experience malnutrition preoperatively [7]. Malnutrition is known to adversely affect postoperative complications and prognosis after TAVI, and its assessment and management are essential [8,9]. In particular, the Geriatric Nutritional Risk Index (GNRI) has been reported to outperform other nutritional indicators in predicting both short- and long-term prognoses for all-cause and cardiovascular mortality following TAVI. This index, calculated from serum albumin (Alb) levels and body weight, is recommended for routine clinical use [8,10]. Additionally, anemia and low hemoglobin (Hb) levels are frequently observed in older patients who have undergone TAVI and have multiple comorbidities preoperatively. These conditions are considered frailty indicators and have been reported to influence mortality from the early to long-term postoperative periods. Therefore, preoperative intervention is warranted [11]. Although comprehensive frailty assessments that incorporate physical and cognitive functions are recognized as important for predicting late bleeding events in patients undergoing TAVI patients [12], their practical application in routine clinical settings remains challenging. A major limitation of such composite indices is that they make it difficult to evaluate the independent prognostic impact of individual components, including anemia and nutrition. This highlights the need to validate simpler indices that specifically combine these two crucial biological markers.

Recently, Wang et al. [13] reported that the Hb-GNRI (H-GNRI), an index based on a combination of Hb levels and GNRI, was associated with prognosis in patients with cancer. This finding suggests the H-GNRI may also serve as a prognostic factor in patients undergoing TAVI, given the high prevalence of malnutrition and anemia in these patients. In particular, clarifying the association between the H-GNRI and MACEs after TAVI is important for guiding postoperative intervention strategies. Furthermore, previous studies on patients with TAVI have failed to establish unified cutoff values for both the GNRI and Hb levels [8,11]. Notably, recent large-scale studies in Japanese patients who underwent TAVI suggest that population-specific cutoff values for both anemia and nutritional status are critical for accurate prognostic prediction [7,14]. In this study, we aimed to investigate the association between H-GNRI and the incidence of MACEs in patients undergoing TAVI, using optimal disease-specific cutoff values determined for our cohort.

## 2. Materials and Methods

### 2.1. Study Design and Participants

**Figure 1:**
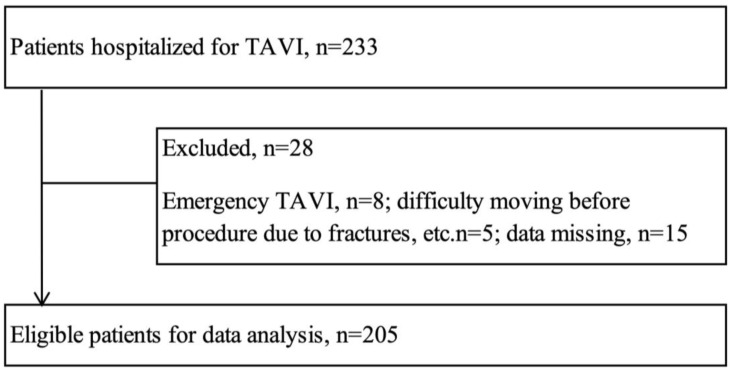
Study flowchart. TAVI, transcatheter aortic valve implantation.

This was a single-center, retrospective, observational study. The study protocol complied with the principles outlined in the Declaration of Helsinki and was approved by the Ethics Committee of the Tohoku University Graduate School of Medicine (2024-1-551) and the Ethical Review Committee of Yamagata University Faculty of Medicine (2025-122). Study participants were patients with AS who were admitted to Yamagata University Hospital as candidates for TAVI and who underwent surgery between May 2017 and April 2024. Exclusion criteria were extreme mobility limitations due to orthopedic diseases, emergency surgery precluding complete preoperative evaluation, and incomplete data. Figure 1 presents a flowchart illustrating the patient selection process. Information about the study implementation was made public on the website to ensure that the study participants or their proxies had the opportunity to refuse participation.

### 2.2. Data Collection

#### 2.2.1. Clinical Variables, Functional Assessments, and Definitions

Using the electronic medical record system, we investigated patient clinical background factors, including age, sex, body mass index (BMI), New York Heart Association (NYHA) functional class, the Society of Thoracic Surgeons predicted risk of mortality score (STS–PROM score) [15], echocardiographic findings (ejection fraction, aortic valve area, aortic peak velocity, aortic valve mean pressure gradient), laboratory data, comorbidities, procedure time, procedure approach route, and length of hospital stay after TAVI. Additionally, a comprehensive geriatric assessment was conducted. Cognitive function was assessed using the Mini-Mental State Examination [16]. Basic activities of daily living (BADL) were assessed using the Katz Index [17], a decline in BADL was defined as requiring assistance in at least one of the six domains. Muscle strength was assessed using the handgrip strength and Short Physical Performance Battery (SPPB) scores [18], and patients with an SPPB score of ≤9 were classified as frail [19].

#### 2.2.2. GNRI

The GNRI, as reported by Bouillanne [20], is a widely used nutrition-related risk indicator calculated from serum albumin level and BMI using the following formula [21]:

GNRI = 14.89 × Alb (g/dL) + 41.7 × current weight/[(height)2 (m) × 22] = 14.89 × Alb (g/dL) + 41.7 × BMI/22

#### 2.2.3. H-GNRI

The H-GNRI, first reported by Wang et al. [13], has been used to classify patients into three groups based on serum Hb levels and the GNRI [20]. The low-risk group (H-GNRI score, 2) included patients with normal Hb levels and a normal GNRI. The intermediate-risk group (H-GNRI score, 1) included patients with low Hb levels or low GNRI. The high-risk group (H-GNRI score, 0) included patients with low Hb levels and low GNRI. In this study, the optimal cutoff values for the hemoglobin level and GNRI were determined using the X-tile program [22], similar to the approach employed by Wang et al. [13] and patients were classified subsequently.

#### 2.2.4. Definition of MACEs

The primary outcome of this study, MACEs, was defined as postoperative death, myocardial infarction, cerebral infarction, severe vascular complications (aortic dissection/rupture, notable bleeding due to arterial injury), and rehospitalization for heart failure [4].

#### 2.2.5. Statistical Analysis

For the patient cohort, continuous and ordinal variables are presented as medians and interquartile ranges (IQRs), and nominal variables as frequencies and percentages. Optimal cutoff values for the Hb level and GNRI were determined using the X-tile program [22], with patients subsequently classified into three groups according to the H-GNRI. Comparisons among the three groups were performed using the χ2 test for nominal variables, and one-way analysis of variance or the Kruskal–Wallis test for continuous variables. The association between H-GNRI and long-term MACEs after TAVI was analyzed using Kaplan–Meier survival curves, and significant differences between groups were assessed using the log-rank test. Factors associated with long-term MACEs after TAVI were identified using univariate and multivariate analyses with a Cox proportional-hazard model. Data analysis was performed using JMP Pro version 17 (JMP Statistical Discovery LLC, Cary, NC, USA), with *p* < 0.05 deemed statistically significant.

## 3. Results

### 3.1. Patient Characteristics

Of the 233 patients who underwent TAVI, 205 were included in the study after excluding 28 patients, eight who underwent emergency procedures, five with severe mobility limitations due to fractures, and 15 with incomplete data. The median age of the patients was 83 years, and 35.2% (*n* = 67) were male (Table 1). The STS–PROM scores indicated a moderate risk of mortality, and echocardiography revealed severe AS. Among the comorbidities, hypertension was the most prevalent (155 patients, 75.2%), followed by dyslipidemia (109 patients, 52.9%), anemia (36 patients, 17.5%), and arrhythmia (59 patients, 28.6%).

### 3.2. H-GNRI Cutoff Values

**Figure 2:**
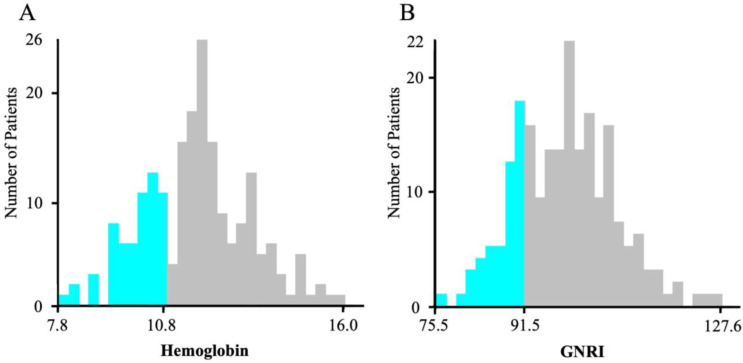
Optimal hemoglobin and GNRI cutoff value. (**A**) Hemoglobin distribution and cutoff value; (**B**) GNRI distribution and cutoff value. GNRI, Geriatric Nutritional Risk Index.

Using the X-tile program, the cutoff value for Hb was set at 10.8 mg/L, with 153 patients assigned to the high Hb group (Hb ≥ 10.8) and 52 patients to the low Hb group (Hb < 10.8). The cutoff GNRI value was calculated as 91.5, with 160 patients assigned to the high GNRI group (GNRI ≥ 91.5) and 45 to the low GNRI group (GNRI < 91.5) (Figure 2).

### 3.3. Comparison of the Three Groups on the Basis of H-GNRI

Based on H-GNRI scores, patients were classified as low-risk (score two: *n* = 123, 60.0%), intermediate-risk (score one: *n* = 67, 32%), and high-risk (score zero: *n* = 16, 8%) (Table 2). Significant differences among the three groups were observed in the incidence of MACEs (*p* = 0.0026), BMI (*p* < 0.0001), proportion of cases showing NYHA functional class 3/4 (*p* = 0.004), STS-PROM score (*p* = 0.0003), GNRI (*p* < 0.0001), Alb level (*p* < 0.0001), Hb level (*p* < 0.0001), and length of hospital stay after TAVI (*p* = 0.0002).

### 3.4. Kaplan–Meier Survival Curves for MACEs Using the Hb Level, GNRI, and H-GNRI

**Figure 3:**
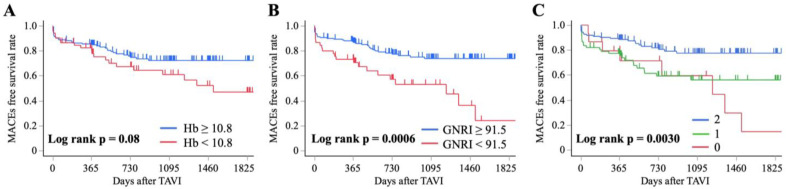
MACEs’ free survival rate according to the cutoff values of Hb, GNRI, and H-GNRI score: (**A**) Hb, (**B**) GNRI, (**C**) H-GNRI. MACEs, major adverse cardiovascular events; Hb, hemoglobin; GNRI, Geriatric Nutritional Risk Index; H-GNRI, hemoglobin-Geriatric Nutritional Risk Index.

Figure 3 presents the Kaplan–Meier survival curves for MACEs according to the Hb level, GNRI, and H-GNRI. The median follow-up period after TAVI was 723.0 days (IQR, 365.5–1267.5 days). MACEs occurred in 57 patients (27.8%), including heart failure in 21 (36.8%), major vascular events in 15 (26.3%), cardiovascular death in 11 (19.3%), cerebrovascular events in nine (15.8%), and myocardial infarction in one (1.8%). The incidence of MACEs did not differ significantly between the high and low Hb groups (log-rank *p* = 0.08; Figure 3A), although a trend toward a higher incidence was observed in the low Hb group (24.1% vs. 38.5%, *p* = 0.047). In contrast, a significant difference was observed between the high and low GNRI groups (log-rank *p* = 0.0006; Figure 3B), with a higher incidence of MACEs in the low GNRI group (22.5% vs. 46.7%, *p* = 0.0014). For the H-GNRI, the incidence of MACEs differed significantly among the three groups (*p* = 0.0026), with a significantly higher rate observed in the intermediate- and high-risk groups compared with the low-risk group (log-rank *p* = 0.0030; score one vs. score two, *p* = 0.0032; score zero vs. score two, *p* = 0.0077; Figure 3C).

### 3.5. Factors Associated with MACEs Using Cox Proportional-Hazard Model: Univariate and Multivariate Analyses

In the univariate analysis, BMI (*p* = 0.03), Hb level (*p* = 0.03), GNRI (*p* = 0.006), H-GNRI (*p* = 0.0063), procedure time (*p* < 0.0001), approach route (*p* = 0.014), and length of hospital stay (*p* < 0.0001) were identified as significant factors. In the multivariate analysis adjusted for age, NYHA classification and SPPB ≤ 9, H-GNRI score (*p* = 0.024; using H-GNRI score two as baseline; H-GNRI score one: hazard ratio [HR] = 2.02, 95% confidence interval [CI]: 1.10–3.60, *p* = 0.021; H-GNRI score zero: HR = 2.67, 95% CI: 1.10–6.44, *p* = 0.028), procedure time (HR = 1.00, 95% CI: 1.00–1.01, *p* = 0.0093), and length of hospital stay after TAVI (HR = 1.02, 95% CI: 1.01–1.04, *p* = 0.0003) (Table 3).

## 4. Discussion

In this study, we observed that preoperative H-GNRI was markedly associated with postoperative MACEs in patients who underwent TAVI. Compared with the low-risk H-GNRI group, the intermediate- and high-risk groups demonstrated a significantly higher risk of MACEs. Furthermore, the cutoff values for Hb and GNRI in this study population differed from previously reported reference values [13,23], underscoring the importance of establishing disease-specific cutoffs tailored to pathological conditions. These findings are consistent with those of previous studies highlighting the prognostic relevance of Hb levels and GNRI individually, while also demonstrating the utility of the H-GNRI as a composite index.

Studies by Jiménez–Xarrié et al. [11] have highlighted the influence of preoperative anemia and reduced Hb levels on the outcomes of TAVI in relation to short- and medium-term prognoses, whereas Baştuğ et al. [24] reported their associations with the long-term prognosis. In contrast, the GNRI has been widely used as a prognostic predictor for older patients and those with heart failure [25]. For example, Ishizu et al. [7] reported that a reduced GNRI was associated with cardiovascular death in Japanese patients who underwent TAVI. Furthermore, a recent meta-analysis suggested that the GNRI is particularly more effective than other nutritional indicators in predicting prognosis [8]. Compared with these previous studies, the current study is novel in demonstrating a strong association between the H-GNRI, which comprehensively evaluates both Hb level and GNRI, and prognostic prediction. In particular, the significant association between the H-GNRI and mortality and non-fatal adverse events, such as MACEs, is consistent with previously reported associations with a decrease in Hb levels, anemia, and nutritional indicators. The H-GNRI used in the current study has previously been shown to be useful in patients with esophageal squamous cell carcinoma and heart failure [13,26]; however, this study differs from prior reports in applying the index to patients with distinct disease backgrounds undergoing TAVI. To the best of our knowledge, the ability of the H-GNRI to predict cardiovascular events, including MACEs, represents novel evidence that expands its potential clinical applications. Numerous patients undergoing TAVI are older and have multiple comorbidities, with several factors contributing to the risk of postoperative cardiovascular events. The H-GNRI is thought to comprehensively reflect these factors and potentially predict the occurrence of MACEs. Furthermore, a key finding of this study was that the predictive value of the H-GNRI was independent of other comprehensive geriatric assessments, including physical frailty, BADL, and cognitive function. This suggests that H-GNRI is not merely a surrogate for a patient’s overall functional status but provides unique prognostic information based on the specific biological states of anemia and malnutrition.

To accurately evaluate the prognostic utility of the H-GNRI in our specific cohort, appropriate cutoff values must be applied. Pre-existing generic criteria, such as the World Health Organization (WHO) definition for anemia, may not be optimal, a notion supported by recent large-scale studies in Japanese patients undergoing TAVI. For instance, Onoda et al. demonstrated that a population-specific Hb cutoff was superior to the WHO definition for predicting mortality [14]. Likewise, the prognostic value of GNRI has been well-established in this population by Ishizu et al. [7]. Therefore, our data-driven approach using the X-tile program was necessary to ensure the robustness of our primary analysis of the predictive value of H-GNRI. Regarding cutoff values that demonstrated an association with MACEs in this study, reports examining the prognosis after TAVI frequently employed the WHO definition for anemia and Hb reduction (males, 13.0 g/dL; females, 12.0 g/dL) [11,27]. However, Nuis et al. [28] reported a poor correlation between the WHO definition and mortality, with a 2.49-fold higher 1-year mortality risk in the group with Hb levels of 10–11 g/dL, which supports the Hb cutoff value identified in this study. This low value may be attributable to the fact that patients undergoing TAVI are often older with numerous comorbidities, such as diabetes, renal dysfunction, and heart failure. These conditions are often associated with chronic inflammation, which in turn is linked to anemia, and have been shown to independently affect prognosis [11,29]. In contrast, Shibata et al. [30] performed a large-scale multicenter study in Japan and found that patients with a GNRI < 92 had a substantially higher 1-year mortality rate, underscoring its value as a prognostic predictor. This cutoff value closely approximates the cutoff value identified in the present study.

Wang et al. [13] and Tohyama et al. [26] demonstrated the usefulness of the H-GNRI using the same cutoff values; however, applying these cutoff values to different patient backgrounds has limitations. For example, a meta-analysis of the GNRI in patients undergoing TAVI indicated the heterogeneity in cutoff values [8]. Similarly, although the WHO definition [27] is commonly used to define anemia, as mentioned above, more sensitive cutoff values tailored to the characteristics of patients undergoing TAVI may be required. In this study, the cutoff values for the Hb level and GNRI were determined to be 10.8 mg/dL and 91.5, respectively, using the X-tile program. X-tile can objectively and statistically derive cutoff values based on survival analysis and is suitable for disease-specific stratification, as it can determine the boundary that yields the most significant survival difference for any continuous variable [22]. In the current study, the cutoff was defined as the point that maximized the log-rank test value in the Kaplan–Meier analysis of MACEs incidence, a method considered statistically valid.

Despite the importance of comprehensive frailty assessments that incorporate physical and cognitive functions, such as the toolset proposed by Tsujimoto et al. [12], their practical application in routine clinical settings can be challenging. A key finding of our study is that the H-GNRI, a relatively simpler index, provides important prognostic information independent of broader geriatric assessments. Nutritional risk discrimination using H-GNRI is a simple method that can be performed using only blood tests and body weight measurements, without requiring specialized equipment. Our findings suggest that H-GNRI could serve as a practical tool to identify patients at higher risk for MACEs who may benefit from further clinical attention or targeted preoperative interventions. Future research should focus on re-validation through prospective multicenter intervention studies to prevent MACEs by addressing preoperative anemia and malnutrition, re-evaluating cutoff values, and constructing integrated risk scores with new indicators.

Our study has several limitations. First, this was a single-center, retrospective observational study, which inherently limits the ability to establish a causal relationship between 2-GNRI and clinical outcomes. Second, given that the study was conducted at a single institution in Japan, our findings may not be generalizable to other populations or healthcare settings. Third, the relatively small sample size may have limited statistical power. Furthermore, traditional frailty indices, such as SPPB, did not demonstrate a significant association with outcomes in our cohort. This may be because our study population, which excluded patients with severe mobility issues before the procedure, lacked a sufficient number of severely frail patients, potentially limiting the statistical power of these functional assessments. Finally, although we adjusted for a wide range of variables, the possibility of unmeasured confounding factors cannot be completely excluded. Accordingly, large-scale, multicenter, prospective studies are required to confirm our findings.

## 5. Conclusions

This study demonstrated that the H-GNRI derived using preoperative Hb level and GNRI was significantly associated with the incidence of postoperative MACEs in patients undergoing TAVI. Our findings suggest that H-GNRI could serve as a simple and valuable tool for risk stratification in patients undergoing TAVI. Notably, this association was independent of other comprehensive geriatric assessments, including physical frailty, BADL, and cognitive function, highlighting its unique value as a simple and objective prognostic marker.

## Figures and Tables

**Table 1:** Patient characteristics.

Variables	Overall (*n* = 205)
Demographics	
Male sex, *n* (%)	67 (35.2)
Age, years	83.0 (80.0–87.0)
BMI, kg/m^2^	22.4 (20.4–25.1)
NYHA functional class 3/4 (%)	87 (42.4)
Surgical risk score	
STS–PROM score, %	5.0 (3.3–7.1)
Echocardiographic data	
Ejection fraction, %	65.0 (60.0–71.0)
Aortic valve area, cm^2^	0.60 (0.50–0.77)
Aortic peak velocity (m/s)	4.3 (4.0–4.8)
Aortic valve mean pressure gradient, mmHg	43.6 (35.7–55.5)
Nutrition	
GNRI score	99.2 (91.8–104.4)
Preprocedural laboratory data	
Hb level, g/dL	11.8 (10.6–12.7)
Alb level, g/dL	3.7 (3.5–4.0)
eGFRcys, mL/min/1.73 m^2^	59.7 (48.1–69.0)
BNP level, pg/mL	93.1 (47.7–286.9)
Physical function	
Grip power, kg	19.7 (15.6–24.1)
SPPB score	12 (9–12)
SPPB ≤ 9, *n* (%)	61 (29.7)
Cognitive function MMSE score	26 (24–28)
BADL dependency (Katz Index)	
Dependent (≥1 item), *n* (%)	28 (13.6)
Comorbidity	
Hypertension, *n* (%)	155 (75.2)
Diabetes mellitus, *n* (%)	65 (31.6)
Dyslipidemia, *n* (%)	109 (52.9)
Atrial fibrillation, *n* (%)	56 (27.2)
Anemia, *n* (%)	36 (17.5)
Chronic kidney disease, *n* (%)	50 (24.3)
Cardiovascular disease, *n* (%)	53 (25.7)
Arrhythmia, *n* (%)	59 (28.6)
Chronic heart failure, *n* (%)	45 (21.8)
Cerebral infarction, *n* (%)	29 (14.1)
Procedural variables	
Procedure time, min	104.0 (90.0–121.8)
Approach route	
Transfemoral approach, *n* (%)	196 (95.1)
Transapical approach, *n* (%)	5 (2.4)
Transaortic approach, *n* (%)	2 (1.0)
Trans-subclavian approach, *n* (%)	2 (1.0)
Length of hospital stay after TAVI, days	11.0 (11–16.5)

Values are numbers (%) or median (95% confidence interval). BMI, body mass index; NYHA, New York Heart Association; STS–PROM, The Society of Thoracic Surgeons predicted risk of mortality; GNRI, Geriatric Nutritional Risk Index; Hb, hemoglobin; Alb, albumin; eGFRcys, cystatin-based estimated glomerular filtration rate; BNP, brain natriuretic peptide; SPPB, Short Physical Performance Battery; MMSE, Mini-Mental State Examination; BADL, basic activities of daily living.

**Table 2:** Relationship between the hemoglobin-Geriatric Nutritional Risk Index score and clinicopathological characteristics (*n* = 205).

Factor	H-GNRI Score			
	2 (*n* = 123)	1 *(n* = 67)	0 (*n* = 15)	*p*-Value
MACEs, *n* (%)	24 (19.5)	25 (37.3)	8 (53.3)	0.0026
Variables				
Male sex, *n* (%)	41 (33.6)	20 (30.0)	6 (40.0)	0.70
Age, years	83.0 (80.0, 86.0)	84.0 (82.0, 88.0)	85.0 (78.0, 87.0)	0.06
BMI, kg/m^2^	23.6 (21.3, 26.1)	21.0 (19.0, 23.8)	20.4 (19.0, 21.5)	<0.0001
NYHA functional class 3/4 (%)	41 (33.3)	39 (58.2)	7 (46.7)	0.004
STS-PROM score, %	4.2 (3.0, 6.4)	5.89 (4.1, 8.10)	7.2 (5.3, 7.2)	0.0003
Echocardiographic data				
Ejection fraction, %	63.8 (62.0, 65.8)	64.1 (61.5, 66.7)	58.5 (53.1, 64.0)	0.14
Aortic valve area, cm^2^	0.67 (0.54, 0.77)	0.60 (0.46, 0.76)	0.65 (0.53, 0.72)	0.15
Aortic peak velocity, m/s	4.2 (4.0, 4.7)	4.5 (4.0, 4.8)	4.4 (4.0, 4.8)	0.30
Aortic valves mean pressure	41.2 (35.6, 54.4)	46.5 (35.4, 58.1)	46.4 (37.3, 56.0)	0.21
gradient, mmHg				
Nutrition				
GNRI score	101.5 (97.3, 106.9)	92.2 (89.6, 100.7)	87.7 (84.1, 89.6)	<0.0001
Preprocedural laboratory data				
Hb level, g/dL	12.1 (11.7, 13.3)	10.5 (10.0, 11.7)	9.7 (8.9, 10.3)	<0.0001
Alb level, g/dL	3.9 (3.6, 4.1)	3.7 (3.5, 3.9)	3.3 (3.0, 3.4)	<0.0001
eGFRcys, mL/min/1.73 m^2^	53.9 (43.3, 64.0)	47.7 (36.7, 58.1)	42.1 (30.7, 47.2)	0.0009
BNP level, pg/mL	62.7 (39.7, 185.4)	141.1 (71.0, 393.6)	98.0 (69.7, 369.8)	0.0002
Physical function				
Grip power, kg	20.0 (16.2, 24.2)	18.7 (14.0, 24.2)	19.4 (12.0, 22.5)	0.20
SPPB, score	12 (9, 12)	11 (8, 12)	9.5 (7.5, 12)	0.26
SPPB ≤ 9, *n* (%)	33 (27.1)	21 (31.3)	7 (50.0)	0.19
Cognitive function				
MMSE	26.0 (25.0, 29.0)	26.0 (24.0, 28.0)	25.0 (21, 27)	0.03
BADL dependency (Katz Index)				
Dependent (≥1 item) *n* (%)	12 (9.8)	14 (20.9)	2 (14.3)	0.10
Procedural variables				
Procedure time, min	103.5 (88.8, 120.0)	104 (90.7, 122.8)	109.0 (92.0, 118.0)	0.74
Approach route				
Transfemoral approach, *n* (%)	120 (98.0)	62 (93.0)	14 (93.0)	0.15
Transapical approach, *n* (%)	0 (0.0)	4 (6.0)	1 (6.7)	
Transaortic approach, *n* (%)	1 (0.8)	1 (1.5)	0 (0.0)	
Trans-subclavian approach, *n* (%)	2 (1.6)	0 (0.0)	0 (0.0)	
Postprocedural variables				
Length of hospital stay after TAVI, days	11.0 (11.0, 13.2)	14.0 (11.0, 18.0)	11.0 (10.0, 18.0)	0.0002

Values are numbers (%) or median (interquartile range). H-GNRI, hemoglobin-GNRI; MACE, major adverse cardiovascular event; BMI, body mass index; NYHA, New York Heart Association; STS–PROM, Society of Thoracic Surgeons predicted risk of mortality; GNRI, Geriatric Nutritional Risk Index; Hb, hemoglobin; Alb, albumin; eGFRcys, cystatin-based estimated glomerular filtration rate; BNP, brain natriuretic peptide; SPPB, Short Physical Performance Battery; MMSE, Mini-Mental State Examination; BADL, basic activities of daily living; TAVI, transcatheter aortic valve implantation.

**Table 3:** Cox proportional-hazard model of the clinical characteristics of MACEs using univariable and multivariable analysis in patients who underwent TAVI.

Factor	Univariate Analysis			Multivariate Analysis		
	HR	95% CI	*p*-Value	HR	95% CI	*p*-Value
Sex Male (Reference)	1.00	-	-			
Female	0.68	0.40–1.16	0.16			
Age	1.03	0.97–1.09	0.31	0.99	0.94–1.06	0.99
BMI	0.92	0.84–0.99	0.03			
NYHA functional class 3/4	1.13	0.66–1.90	0.64	1.27	0.40–1.39	0.36
SPPB ≤ 9	0.77	0.43–1.41	0.41	0.75	0.40–1.39	0.36
MMSE	1.04	0.95–1.15	0.37			
BADL Dependent (≥1 item)	0.48	0.17–1.35	0.16			
Hb level	0.83	0.70–0.99	0.03			
GNRI	0.96	0.92–0.98	0.006			
H-GNRI score (baseline, H-GNRI score of 2)			0.0063			0.024
H-GNRI score = 1	2.25	1.28–3.95	0.004	2.02	1.10–3.60	0.021
H-GNRI score = 0	2.85	1.26–6.40	0.011	2.67	1.10–6.44	0.028
Procedure time	1.01	1.00–1.01	<0.0001	1.00	1.00–1.01	0.0093
Transfemoral approach (for Transapical)	0.27	0.10–0.77	0.014	0.49	0.16–1.47	0.66
Length of hospital stay after TAVI	1.03	1.02v1.04	<0.0001	1.02	1.01–1.04	0.0003

HR, hazard ratio; CI, confidence interval. BMI, body mass index; NYHA, New York Heart Association; SPPB, Short Physical Performance Battery; MMSE, Mini-Mental State Examination; Hb, hemoglobin; GNRI, Geriatric Nutritional Risk Index; H-GNRI, hemoglobin-Geriatric Nutritional Risk Index; BADL, basic activities of daily living; TAVI, transcatheter aortic valve implantation. Not used for multivariable analysis owing to collinearity issues.

## Data Availability

The datasets generated and analyzed during the current study are not publicly available due to ethical restrictions (e.g., containing potentially identifiable patient information), but are available from the corresponding author upon reasonable request.

## Abbreviations

The following abbreviations are used in this manuscript:

TAVI	Transcatheter aortic valve implantation
AS	Aortic stenosis
MACEs	Major adverse cardiovascular events
GNRI	Geriatric Nutritional Risk Index
Alb	Serum albumin
Hb	Hemoglobin
H-GNRI	Hemoglobin-GNRI
BMI	Body mass index
NYHA	New York Heart Association
STS-PROM	Society of Thoracic Surgeons Predicted Risk of Mortality
SPPB	Short Physical Performance Battery
BADL	Basic activities of daily living
HR	Hazard ratio
CI	Confidence interval
IQR	Interquartile ranges
WHO	World Health Organization
